# Estrogen Receptor-Targeted Contrast Agents for Molecular Magnetic Resonance Imaging of Breast Cancer Hormonal Status

**DOI:** 10.3389/fonc.2016.00100

**Published:** 2016-04-27

**Authors:** Adi Pais, Hadassa Degani

**Affiliations:** ^1^Department of Biological Regulation, Weizmann Institute of Science, Rehovot, Israel

**Keywords:** estrogen receptor-targeted probes, contrast agents for MRI, breast cancer, estrogen receptor, molecular imaging

## Abstract

The estrogen receptor (ER) α is overexpressed in most breast cancers, and its level serves as a major prognostic factor. It is important to develop quantitative molecular imaging methods that specifically detect ER *in vivo* and assess its function throughout the entire primary breast cancer and in metastatic breast cancer lesions. This study presents the biochemical and molecular features, as well as the magnetic resonance imaging (MRI) effects of two novel ER-targeted contrast agents (CAs), based on pyridine-tetra-acetate-Gd(III) chelate conjugated to 17β-estradiol (EPTA-Gd) or to tamoxifen (TPTA-Gd). The experiments were conducted in solution, in human breast cancer cells, and in severe combined immunodeficient mice implanted with transfected ER-positive and ER-negative MDA-MB-231 human breast cancer xenografts. Binding studies with ER in solution and in human breast cancer cells indicated affinities in the micromolar range of both CAs. Biochemical and molecular studies in breast cancer cell cultures showed that both CAs exhibit estrogen-like agonistic activity, enhancing cell proliferation, as well as upregulating cMyc oncogene and downregulating ER expression levels. The MRI longitudinal relaxivity was significantly augmented by EPTA-Gd in ER-positive cells as compared to ER-negative cells. Dynamic contrast-enhanced studies with EPTA-Gd *in vivo* indicated specific augmentation of the MRI water signal in the ER-positive versus ER-negative xenografts, confirming EPTA-Gd-specific interaction with ER. In contrast, TPTA-Gd did not show increased enhancement in ER-positive tumors and did not appear to interact *in vivo* with the tumors’ ER. However, TPTA-Gd was found to interact strongly with muscle tissue, enhancing muscle signal intensity in a mechanism independent of the presence of ER. The specificity of EPTA-Gd interaction with ER *in vivo* was further verified by acute and chronic competition with tamoxifen. The chronic tamoxifen treatment also revealed that this drug increases the microvascular permeability of breast cancer xenograft in an ER-independent manner. In conclusion, EPTA-Gd has been shown to serve as an efficient molecular imaging probe for specific assessment of breast cancer ER *in vivo*.

## Introduction

Breast cancer is the most common malignancy in women and the second leading cause of cancer death among women (1). The overexpression of the estrogen receptor (ER) (2) is currently an established molecular feature for assessing breast cancer prognosis and predicting response to endocrine therapies [Ref. (3) and references cited therein]. The critical importance of ER measurements in managing breast cancer treatment was recently emphasized in the results of a meta-analysis of randomized trials showing that ER status of the primary tumor was the only patient or tumor characteristic that strongly predicted tamoxifen efficacy, whereas the progesterone receptor measurement did not seem to be importantly predictive of efficacy (4).

Estrogen receptor status is predominantly evaluated today by immunohistochemistry staining of ER, which is a semi-quantitative method and, therefore, may lack reproducibility and standardization across different laboratories (5–8). In addition, this method requires fresh randomly selected tumor tissue, not always available, particularly in metastatic breast disease.

Part of the above described limitations can be overcome by developing molecular imaging techniques that will detect and map ER level over the entire tumor tissue in a quantitative manner. Thus, ER imaging has become an important target for future development (9). Today, molecular imaging of ER is primarily based on the application of radiolabeling selective estrogen receptor modulators (SERMs) that can be detected by single photon emission computed tomography (SPECT) or positron emission tomography (PET) (10). The most clinically advanced ER imaging method today uses 16-α-[f-18]-fluoro-17-β-estradiol (FES) and PET (11). These methods provided quantitative imaging ER expression *in vivo* in animal models and in breast cancer patients (12–14), but it is not applicable yet as a routine imaging technique for the workup of breast cancer patients.

Currently, magnetic resonance imaging (MRI) methods demonstrated excellent efficiency for breast cancer detection and diagnosis [Ref. (15) and references cited therein]. The challenge of molecular MRI to evaluate ER expression can provide a direct critical prognostic factor at the stage of diagnosis. Therefore, we embarked on developing novel contrast agents (CAs) targeted to the ER that should be detected by MRI. To that end, we have synthesized two new CAs targeted to the ER, which are composed of Gadolinium chelate of pyridine-tetra-acetate (PTA-Gd) conjugated with the native ligand 17β-estradiol (EPTA-Gd) or with the antiestrogen tamoxifen (TPTA-Gd), and evaluated their MRI properties in solution, in breast cancer cells and in breast cancer xenografts in animal models (16–18). In addition, direct structural information on the crystal structure of the ligand-binding domain of ER bound to the europium chelate of EPTA (EPTA-Eu) was obtained using X-ray crystallography (19). This paper presents characterization of the binding capacity and the hormonal/molecular effects of EPTA-Gd and TPTA-Gd in human breast cancer cells, as well as restates and expands the data evaluation of the MRI properties of these CAs in cell cultures and animal models of breast cancer. We have focused on investigating the interaction and binding affinity with ER, the hormonal-induced changes in cell proliferation, and the up or downregulation of estrogen-induced genes. Furthermore, investigation of the ER-specific and non-specific interactions of these probes in breast cancer cells and tumors and in muscle tissue, as well as the competition with tamoxifen emphasized the advantage of EPTA-Gd over TPTA-Gd as an ER-targeted CA *in vivo*.

## Materials and Methods

### Solution-Binding Affinity to ER

The binding affinities were measured by a radioactive inhibitory competitive assay in solution, using recombinant hERα (1.76 nM) (PanVera, Inc., Madison, WI, USA), tritiated 17β-estradiol (^3^HE2 = 2.0–3.5 nM, 140 Ci/mmol) (NEN, Boston, MA, USA), and EPTA-Gd and TPTA-Gd as the competing ligands. Experiments were done in duplicates. The concentration of competing ligand required to replace half of the tritiated 17β-estradiol that would be bound to the hERα, IC50, was derived by non-linear regression analysis of the experimental data to the following equation:

$$Y=\text{Non-Specific Binding}+\left(\text{Total Binding}-\text{Specific Binding}\right)/\left(\text{1}+10^{\text{log}X-\text{logIC5}0}\right)$$

with *Y*, the observed data and *X*, the inhibitor concentration; non-specific binding of ^3^HE2 is measured by competition with excess of 1 μM cold E2 and total-specific binding is maximal binding of ^3^HE2 measured without competition. The absolute inhibition constant, Ki, was determined according to the Cheng–Prusoff equation:

$$\text{Ki}=\text{IC50}/[1+{(}^{3}\text{HE2}/\text{Kd})]$$

using Kd = 0.2 nM of 17β-estradiol.

### Cells

T47D (clone 11) and MDA-MB-231 human breast cancer cells were cultured in RPMI 1640 medium supplemented with 10% FCS (Biological Industries, Israel), 4 mM l-glutamine, and 0.1% combined antibiotics (Bio-Lab, Israel). In addition, T47D medium included insulin (0.8 ml/l) and MDA medium included pyruvate (1 mM). MCF7 human breast cancer cells were cultured in DMEM medium supplemented with 6% FCS (Biological Industries, Israel), 4 mM l-glutamine, and 0.1% combined antibiotics (Bio-Lab, Israel).

Estrogen receptor-positive MDA-MB-231 cells were obtained by stably transfecting the wild type (WT) MDA-MB-231 cells with a plasmid encoding tetracycline repressor (TR) protein pcDNA6/TR (T-REX™ System, Invitrogen, USA) and with a plasmid encoding ERα pcDNA4/ER, as previously described (20). The expression of ERα in these cells was induced by adding doxycycline (1 μg/ml) (doxycycline hyclate, Sigma-Aldrich, MO, USA) to the growth medium for at least 3 days.

### Cell Proliferation Assay

Cells were grown in phenol red-free medium supplemented with 10% dextran-coated charcoal stripped fetal bovine serum – DCC-FBS (Biological industries, Beit Haemek, Israel) for a minimum of 5 days and were then seeded in a 96-well plate (3.5 × 10^3^ cells/well) and cultured in the same medium with the various treatments administered to the medium. The number of cells was determined by the cell viability MTT (3-(4,5-dimethylthiazol-2-yl)-2,5-diphenyl tetrazolium bromide) assay (21). Each data point presents an average of six-replicate wells in a single experiment; experiments were repeated several times as indicated in the text.

### Western Blotting

Estrogen receptor α, cMyc, and α-tubulin protein levels were determined using immunoblotting with mouse anti-human ERα antibody (clone 6F11, Novocastra Laboratories, UK), mouse anti-human cMyc antibody (9e10, Abcam, MA, USA), and mouse anti-human α-tubulin antibody (clone DM-1A, Sigma-Aldrich, MO, USA), respectively. Goat anti-mouse horseradish peroxidase and alkaline phosphatase were used as secondary antibodies (Jackson ImmunoResearch Laboratories, PA, USA). Densitometric analyses were performed using Quantity One 4.6 (Bio-Rad Laboratories, CA, USA). The changes in the expression due to treatment with the ER-targeted probes were performed in cells grown in phenol red-free medium for a minimum of 5 days prior to the treatment. The expression of ER in the different human breast cancer cell lines was quantified by normalizing the intensity of the bands to those of standard, known concentrations of recombinant hERα protein (PanVera, Inc., Madison, WI, USA).

### MRI- and Fluorescence-Binding Studies

The interaction of the ER-targeted probes with ER-positive and ER-negative MDA-MB-231 cells were investigated by T1-relaxation measurements and by a fluorescence assay using the Eu-chelate of EPTA, EPTA-Eu. Studies were also performed on cells cultivated on microspheres. The cells, ~3 × 10^6^, were seeded on 0.5 ml microspheres (Biosilon polystyrene microspheres, NUNC, 160–300 μm diameter) placed in non-adherent bacterial Petri dishes using FCS-supplemented DMEM medium. After 4 days, the medium was replaced by phenol red-free medium supplemented with DCC-FBS for additional 3 days. On day 7, the medium was replaced by serum-free medium containing either EPTA-Gd or TPTA-Gd for 60 min incubation and placed in wells (0.5 ml/well) (Microtest 96-well plate, BD Falcon, NJ, USA). Proton T1-relaxation rates were measured at 23°C with a 4.7-T Bruker Biospec spectrometer (Bruker, Karlsruhe, Germany) by applying a 2D spin-echo pulse sequence, field of view (FOV) 8 cm × 8 cm, matrix of 256 × 192, slice thickness of 3 mm, echo time (TE) = 16 ms, and six different repetition times (TRs). T1-relaxation times per pixel were calculated by non-linear least-squares fitting (using simplex algorithm Matlab R2009b, MathWorks, Natick, MA, USA) of the MRI signal intensity, SI, to the equation SI = So[1 − exp(−TR/T1)] with two free parameters So and T1.

T1- and T2-relaxation rate measurements were also conducted in ER-positive and ER-negative cells cultured on microspheres and perfused during the experiments under sterile conditions with oxygenated, phenol red-free, and serum-free medium at 36 ± 1°C, as previously described (22). The measurements were conducted in a 9.4-T NMR spectrometer (DMX-400, Bruker, Karlsruhe, Germany). EPTA-Gd and TPTA-Gd were gradually added at various concentrations (range 0.1–7.5 μM) to the perfusion medium reservoir and at the end of the experiments, the CA was washed out by fresh medium. Proton T1-relaxation rates (R1) and T2-relaxation rates (R2) were measured by MRS of the water signal using standard inversion recovery pulse sequence and a Car–Purcell–Meiboom–Gill sequence, respectively. ΔR1 and ΔR2 were defined as the difference between R1 (or R2) of cells perfused with medium containing the CA and the contrast-free medium. T1 relaxivity, r1, in mM^−1^ s^−1^, was calculated from the slope of a linear fit of ΔR1 as a function of the CA concentration.

For the fluorescence-binding assay, both ER-positive and ER-negative cells were seeded and grown on polystyrene Biosilon beads, as described above, placed in glass Petri dishes covered with silicone (Sigmacote, Sigma-Aldrich, MO, USA) to minimize non-specific binding. On day 7, the medium was replaced with fresh serum-free medium containing EPTA-Eu at concentrations ranging from 0.1 to 0.5 μM for 60 min at 37°C. Then, the cells were washed three times with 10 ml of the fresh medium, and DELFIA enhancement solution (1244-105, PerkinElmer, MA, USA) was added (4–5 ml/plate) and stirred in the dark for 15 min at room temperature. The solution was then transferred into a 48-well plate (300 μl/well in quadruplicate) and scanned on a Wallac Victor3 instrument, using the standard europium time resolved fluorescence measurement (340 nm excitation, 400 μs delay, and emission collection for 400 μs at 615 nm). The specific binding to ER was calculated by subtracting the non-specific fluorescence of ER-negative cells from the fluorescence of ER-positive cells. The fluorescence optical density (OD) intensities were converted to molar units by using a calibration curve obtained from OD values of known EPTA-Eu concentrations. The final concentration data points reflecting specific binding in the cells to ER were fitted to a one-site binding equation *Y* = Bmax × *X*/(Kd + *X*), where *Y* is the measured OD converted to molar units and *X* is the administrated concentration of EPTA-Eu using non-linear least-squares Levenberg–Marquardt algorithm (origin version 6.1) yielding the dissociation constant Kd, which is the inverse of the association constant Ka and maximal-binding capacity (Bmax) of EPTA-Eu to ER.

### MRI of Breast Cancer Xenografts in Mice

All experimental protocols were reviewed and approved by the Institutional Animal Care and Use Committee of the Weizmann Institute of Science. Female CB-17 severe combined immunodeficient (SCID) mice (Harlan Biotech Israel Ltd., Israel), 6–7 weeks old, were ovariectomized. About a week later, WT human MDA-MB-231 breast cancer cells and stable ER-transfected MDA-MB-231 cells were inoculated (2.5 × 10^6^ cells in 0.1 ml phosphate-buffered saline) into the left and right mammary fat pad, respectively. One week later, ER expression in the implanted cells was induced by supplementing the drinking water with 0.2 mg/ml doxycyclin (44577 doxycycline hyclate, Sigma-Aldrich, MO, USA) in 3% sucrose. The size of the xenografts was measured by caliper, estimating the volume by assuming a hemielipsoid shape according to volume = (length/2 × width/2 × height/2) × 4π/3.

*In vivo* MRI experiments were conducted 2–4 weeks after cell implantation. During the MRI scanning, mice were anesthetized with isoflurane (Medeva Pharmaceuticals, Inc., Bethlehem, PA, USA) (3% for induction and 1–2% for maintenance) mixed with compressed air (1 l/min) and delivered through a nasal mask. Once anesthetized, the animals were placed in a head-holder to assure reproducible positioning inside the magnet. Respiration rate was monitored and kept throughout the scanning period around 60–80 breaths per minute.

All *in vivo* MR images were acquired on a 9.4-T Biospec AVANCE II spectrometer (Bruker, Karlsruhe, Germany). The protocol included a multi-slice T2-weighted sequence and a dynamic contrast-enhanced (DCE) 3D gradient-echo pulse sequence with TE/TR 2.5/15 ms and flip angle 40°, four averages (1.5 min). The latter images alternated between images of the tumors recorded in the axial direction with a spatial resolution of 0.156 mm × 0.156 mm × 1.2 mm and images of the descending aorta and muscle tissue in coronal direction with spatial resolution of 0.234 mm × 0.156 mm × 1 mm.

Each ER-targeted probe was injected as a bolus into the tail vein of the mice. The dose of EPTA-Gd was 0.03 mmol/kg (*n* = 4) or 0.075 mmol (*n* = 5). The dose of TPTA-Gd was 0.075 mmol/kg (*n* = 4).

Competition of EPTA-Gd with tamoxifen was tested *in vivo* using two modes of tamoxifen treatment, acute (1 h) and chronic (3 days). In the acute treatment, tamoxifen citrate salt – TAM (T9262, Sigma-Aldrich, St. Louis, MO, USA) dissolved in sterile sunflower oil (4 mg/ml) or 4-hydroxytamoxifen – OHT (H7904, Sigma-Aldrich) dissolved in sterile sunflower oil containing 20% ethanol were stirred overnight at 37°C and administered by intraperitoneal injection at a final dose of 0.07 mmol/kg TAM (*n* = 3) or 0.1 mmol/kg OHT (*n* = 1). One hour later, EPTA-Gd was injected into the tail vein at a dose of 0.075 mmol/kg. In the chronic treatment, tamoxifen pellets (5 mg/pellet, 4w-release time; Innovative Research of America, Sarasota, FL, USA) were implanted subcutaneously in the back of the mouse and 3 days later ETPA-Gd was administered (*n* = 4). The MRI protocol was the same as described above for the direct, non-competitive, contrast-enhanced experiments.

Changes in signal intensity were calculated per pixel yielding enhancement datasets defined as [*I*(*t*)−*I*(0)]/*I*(0), where *I*(0) and *I*(*t*) are the signal intensities pre- and post-contrast, respectively. Enhancement maps at pixel resolution were calculated in regions of interest (ROI) in all slices including tumors’ tissue. ROIs were delineated on the anatomical T2-weighted images and transferred to the corresponding DCE images. *I*(0) per pixel was calculated as a mean intensity of the four pre-contrast images.

### Histology

The tumors were removed, fixed in 4% formaldehyde, sectioned to 4 μm histological slices and stained with hematoxylin and eosin (H&E), as well as immune-stained for nuclear ERα. The immunostaining was performed using rabbit monoclonal anti-ER antibody (ER-SP1, Ventana Medical System, AZ, USA) and an automated slide staining BenchMark XT system operated, according to the manufacturer’s instructions (Ventana Medical System, AZ, USA). An experienced breast pathologist evaluated the extent of intensity of staining [absent (*i* = 0), weak (*i* = +1), moderate (*i* = +2), or strong (*i* = +3)], and the percentage of ER-stained cell nuclei. These two evaluations were used for calculating a specific intensity index defined as: Σ*I* (*i*) × fraction of cells stained with (*i*).

### Statistics

Student’s two-tailed paired *t*-tests (GraphPad Software, Inc., QuickCalcs Web site http://www.graphpad.com/quickcalcs/ttest1.cfm) were applied to evaluate the effect of each treatment on the measured cellular parameter relative to control non-treated cells or to control ER-negative cells undergoing the same treatment. This test was also applied to evaluate the differences between the size and EPTA-Gd-induced enhancement in the ER-positive and ER-negative xenografts. A level at *p* < 0.05 was considered significant.

## Results

### Binding to ER in Solution and in Cells

**Figure 1:**
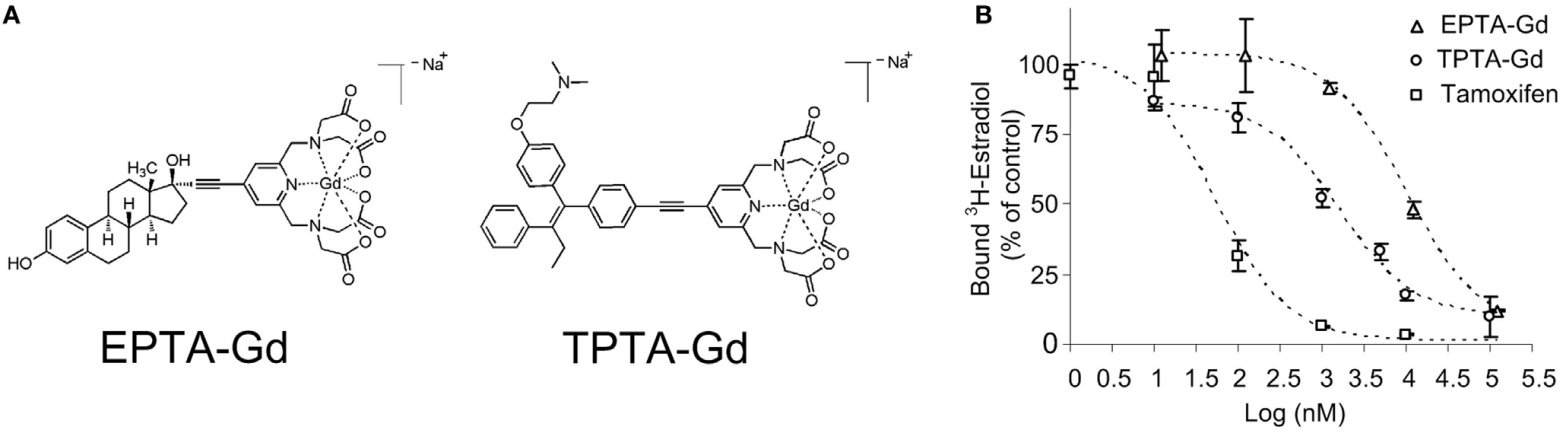
**The chemical structure of 17β-estradiol pyridine-tetra-acetate-Gd (EPTA-Gd) and tamoxifen pyridine-tetra-acetate-Gd (TPTA-Gd) (A) and their competitive displacement curves by tritiated 17β-estradiol (^3^HE2) on a human recombinant ERα in reference to tamoxifen competition (B)**.

The chemical structures of the two gadolinium chelate of pyridine-tetra-acetate (PTA-Gd) conjugated with the native ligand 17β-estradiol (EPTA-Gd) or with the antiestrogen tamoxifen (TPTA-Gd) are presented in Figure 1A. The binding affinities of EPTA-Gd and TPTA-Gd to an isolated hERα were determined in reference to tamoxifen by a competitive radioactive-binding assay with ^3^HE2 as described in Section “Materials and Methods” (Figure 1B). Non-linear least-squares fitting of the experimental data yielded inhibitory dissociation constants Ki_EPTA-Gd_ = 0.97 ± 0.07 μM and Ki_TPTA-Gd_ = 0.13 ± 0.006 μM as compared to Ki_Tamoxifen_ = 0.005 ± 0.001 μM.

Specific binding of these agents to ER in human breast cancer cells was demonstrated by augmentation in the T1-relaxation rate in ER-positive cells as compared to ER-negative cells cultivated on microspheres. The change in T1-relaxation rate in the ER-positive cells in the presence of EPTA-Gd (6 μM), measured in eight independent experiments, yielded a mean ± SD of 74 ± 20 ms^−1^. This change in T1-relaxation rate was significantly higher than that in ER-negative cells of 46 ± 20 ms^−1^ (*p* = 0.02, *n* = 8). Nine independent experiments with TPTA-Gd (5 μM) augmented the T1-relaxation rate in ER-positive cells by a mean ± SD of 72 ± 6 ms^−1^ and in ER-negative cells by 64 ± 10 ms^−1^ with a borderline significant difference between the cells (*p* = 0.07, *n* = 9).

**Figure 2:**
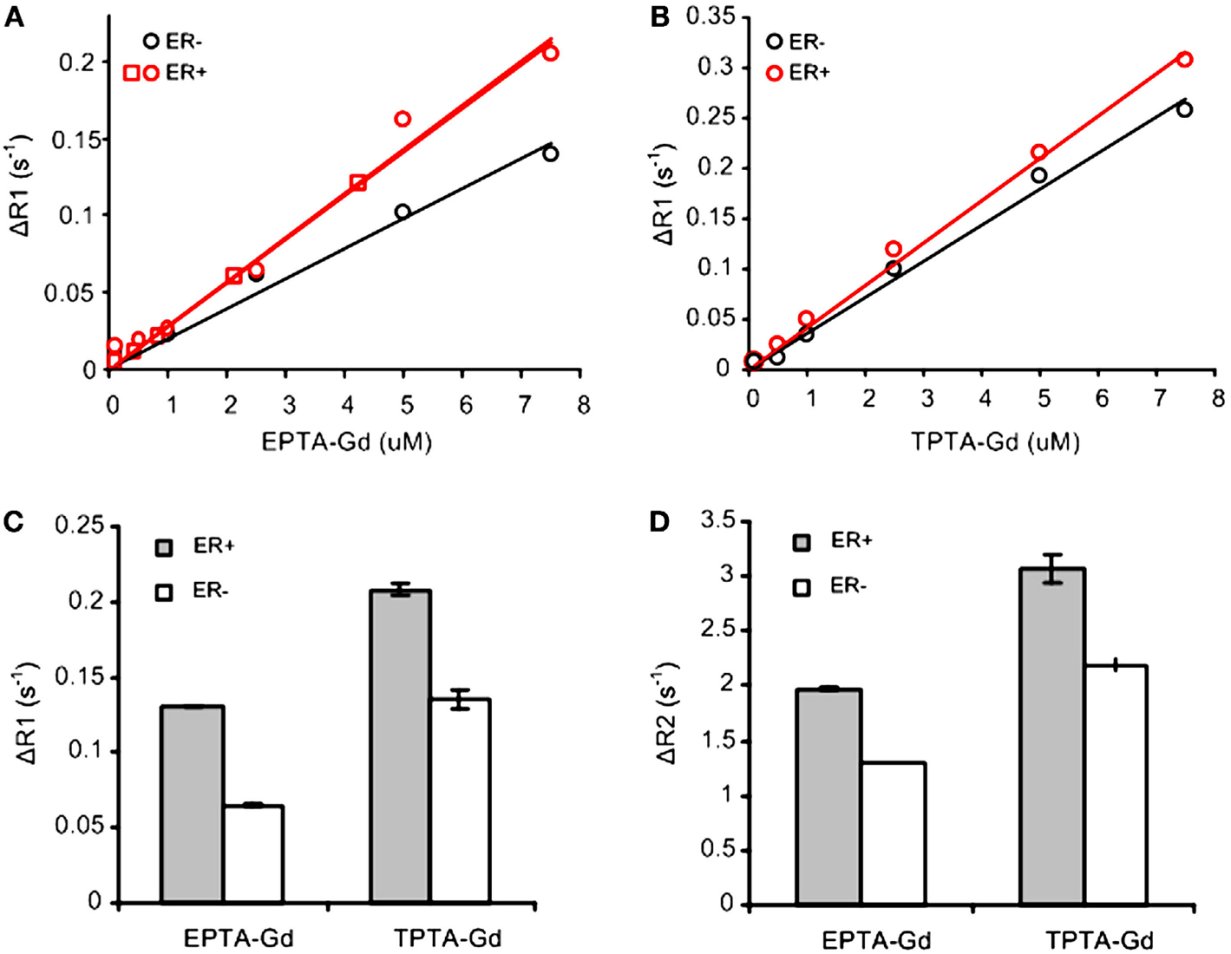
**T1 and T2 measurements with EPTA-Gd and TPTA-Gd in perfused ER-positive and ER-negative MDA-MB-231 human breast cancer cells**. ΔR1 and ΔR2 are defined as the difference between R1 (or R2) of cells perfused with medium containing the contrast agent and R1 (or R2) of these cells perfused with contrast-free medium. **(A)** T1 relaxivity of EPTA-Gd in ER-positive and negative cells: r1 (ER-positive) = 28.5 ± 0.1 mM^−1^s^−1^ (*n* = 2) and r1 (ER-negative) = 19.6 mM^−1^s^−1^. **(B)** T1 relaxivity measurements of TPTA-Gd in ER-positive and negative cells: r1 (ER-positive) = 42.1 mM^−1^s^−1^ and r1 (ER-negative) = 36 mM^−1^s^−1^. The cells were perfused in the NMR tube and treated with increasing concentrations of each contrast agent. T1-relaxation time of water protons was determined at 9.4 T using inversion recovery pulse sequence. Different symbols for ER-positive cells in **(A)** represent two independent experiments. r1 relaxivities were calculated as the slope of the linear fit to the data as explained in the text. **(C)** Change in T1-relaxation rates, ΔR1, in the perfused cells after washing out EPTA-Gd (7.5 μM) or TPTA-Gd (7.5 μM) from the perfusion system with fresh medium. **(D)** Change in T2-relaxation rates, ΔR2, in the perfused cells as in **(C)**. Data presented in **(C,D)** are mean ± SD of three to six measurements recorded 30–60 min after the beginning of the washout process.

Concentration-dependent studies of the T1-relaxation rates (R1) of ER-positive and ER-negative breast cancer cells cultivated on microspheres and perfused during the experiments with increasing concentrations of each CA indicated an increase in T1 relaxivity due to binding to ER (Figures 2A,B). The concentration dependence of ΔR1 (the difference between R1 of cells perfused with medium containing the CA and the same cells perfused with contrast-free medium) showed that EPTA-Gd and TPTA-Gd augment the T1 relaxivity in ER-positive cells as compared to ER-negative cells by 45 and 22%, respectively. After washing out EPTA-Gd (7.5 μM) or TPTA-Gd (7.5 μM) from the perfusion system with fresh medium, both ΔR1 and ΔR2 remained significantly higher in ER-positive cells as compared to ER-negative cells. Generally, gadolinium-based CAs will affect both T1- and T2-relaxation rates and thus the results above confirm specific binding of these agents to ER (Figures 2C,D).

**Figure 3:**
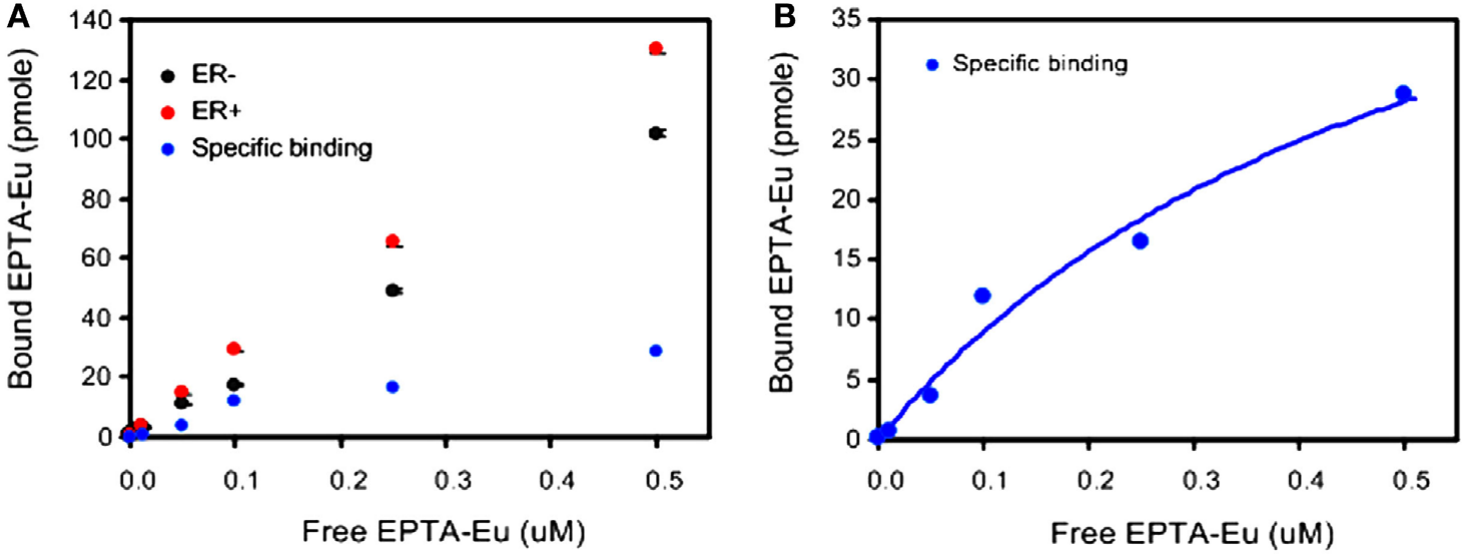
**Binding affinity of EPTA-Eu to ER in human breast cancer cells**. ER-positive and ER-negative MDA-MB-231 human breast cancer cells, grown on microspheres, were incubated in the presence of the indicated concentrations of EPTA-Eu for 1 h at 37°C and subjected to DELFIA assay. **(A)** Total binding (red) was determined in ER-positive cells and non-specific binding (black) was determined in ER-negative cells. Specific binding (blue) was calculated by subtracting non-specific from total binding. **(B)** Saturation-binding curve. Data points of specific binding were fitted to one-site binding equation yielding Kd = 0.56 μM, BMAX = 60.9 pmole, and R2 = 0.97. Data of OD scale were converted to molar units by comparing to OD values of known EPTA-Eu concentrations.

Further verification of the binding to ER was performed by direct measurement of the binding affinity of cellular ER to the europium complex of EPTA-Eu, using a dissociation-enhanced lanthanide fluorescence assay. Specific binding to ER was obtained by subtracting the non-specific binding in ER-negative cells from the total binding in ER-positive cells (Figure 3). The specific binding curve (Figure 3B) yielded an association constant in the cells, Ka_EPTA-Eu_ of 1.75 ± 0.2 μM and maximal-binding capacity Bmax = 3.53 ± 0.17 pmole per 10^6^ cells (*n* = 3).

### Hormonal-Induced Bioactivities of EPTA-Gd and TPTA-Gd in Human Breast Cancer Cells

**Figure 4:**
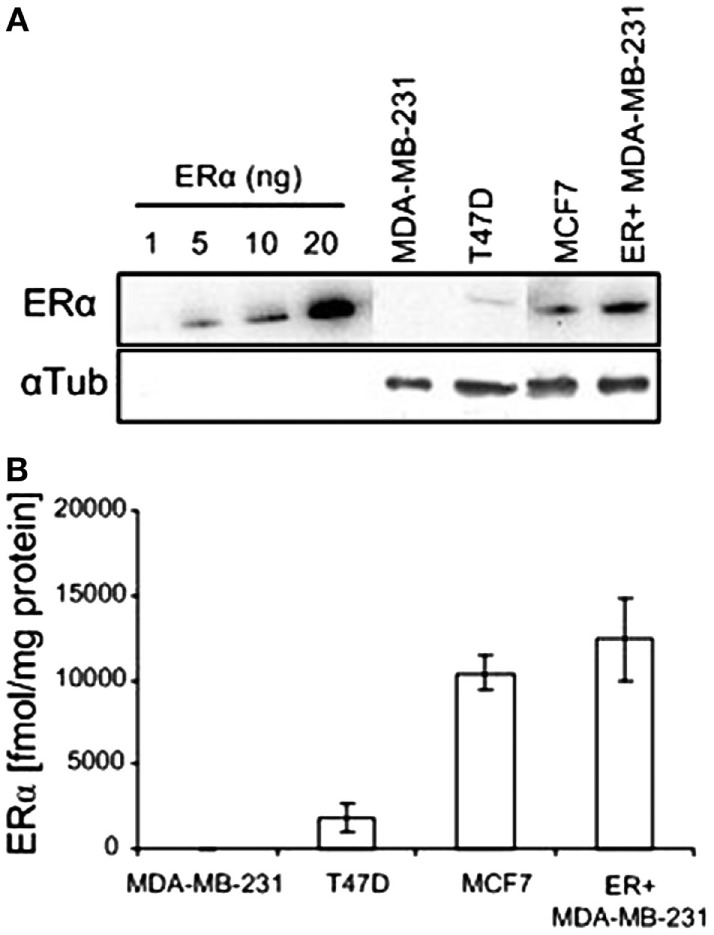
**ERα expression in various human breast cancer cell lines**. **(A)** Western blots of recombinant hERα and of ERα in human breast cancer cell extracts. **(B)** Quantification of western blot experiments (*n* = 2).

The protein level of ERα in the various human breast cancer cells examined in this study were determined by western blotting, calibrating the levels using a calibration curve of commercial hERα protein (Figure 4). The level of ER in the estrogen responsive cells T47D and MCF7 was 1,802 ± 842 and 10,377 ± 1,044 fmol/mg protein, respectively. The WT MDA-MB-231 cells had a null level, and the ER-transfected MDA-MB-231 cells had a high level of 14,800 ± 980 fmol/mg protein. The expression levels in T47D, MCF7, and WT MDA-MB231 cells are in accord with previously published values for these cells (20, 23, 24).

**Figure 5:**
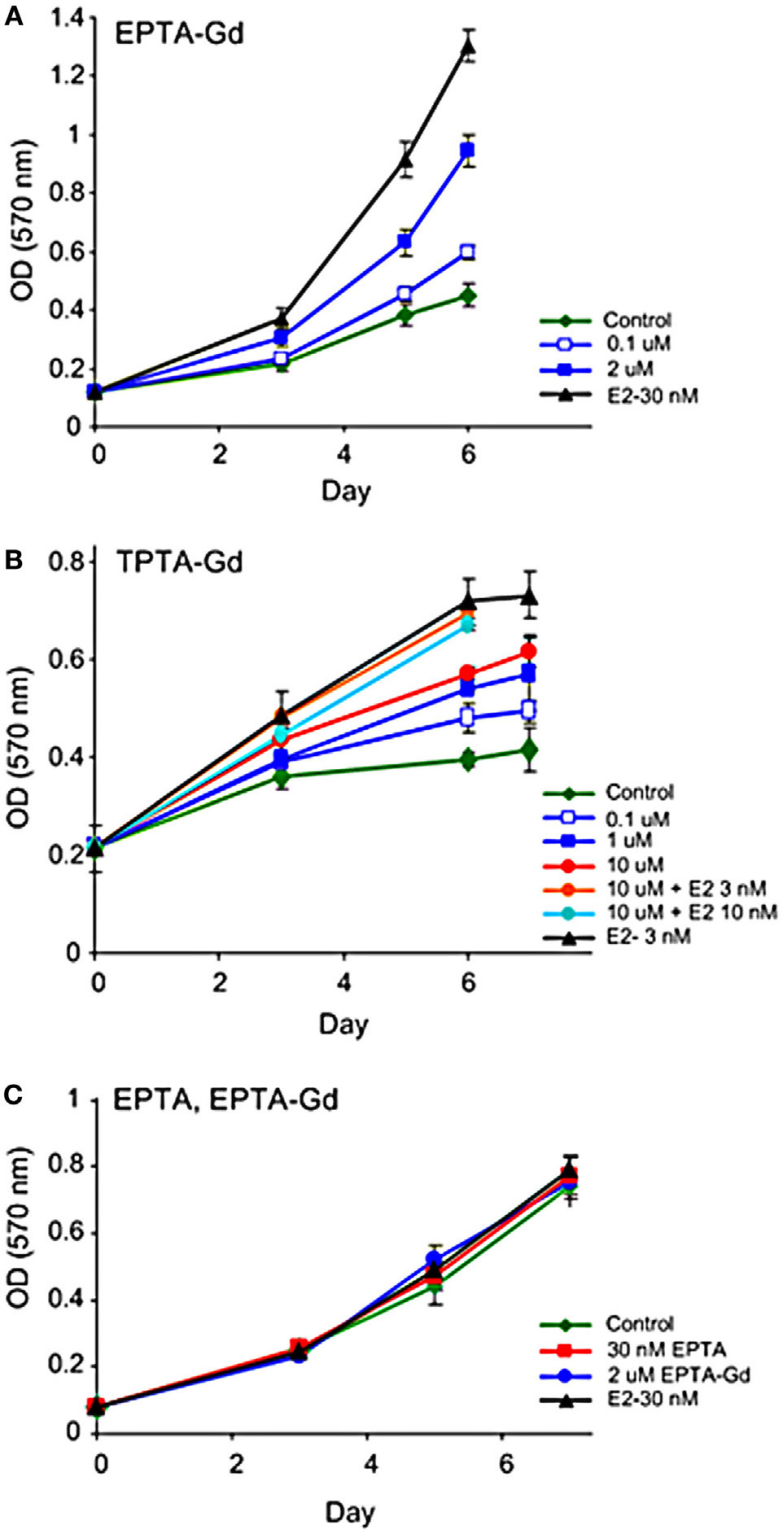
**Effect of EPTA-Gd and TPTA-Gd on the proliferation rates of human breast cancer cells**. **(A)** Dose-dependent effect of EPTA-Gd on the proliferation rate of ER-positive T47D human breast cancer cells. **(B)** Dose-dependent effect of TPTA-Gd and of TPTA-Gd + E2 on the proliferation rate of ER-positive MCF7 human breast cancer cells. **(C)** Dose-dependent effect of EPTA-Gd on the proliferation rate of ER-negative WT MDA-MB-231 human breast cancer cells. Cell proliferation was measured by the cell viability MTT assay, and each data point represents mean ± SD of six-replicate wells. Control: cells cultivated in E2-free medium.

Both EPTA-Gd and TPTA-Gd stimulated the proliferation of T47D and MCF7 cells in a dose- and time-dependent manner, but did not affect the proliferation rate of ER-negative MDA-MB-231 cells (Figure 5). Statistical analysis of the proliferation rate of MCF7 and T47D cells induced by 1–2 μM EPTA-Gd showed a reproducible and significant stimulation of approximately twofold (*p* < 0.02, *n* = 5, two-tail paired *t*-test).

Unlike tamoxifen, competition of TPTA-Gd with E2 did not affect the proliferation of T47D and MCF7 cells (*p* > 0.3, two-tailed paired *t*-test, *n* = 7). Thus, both ER-targeted probes exerted mild agonistic effect on the proliferation of estrogen responsive cells and were neither cytotoxic nor cytostatic to these cells.

**Figure 6:**
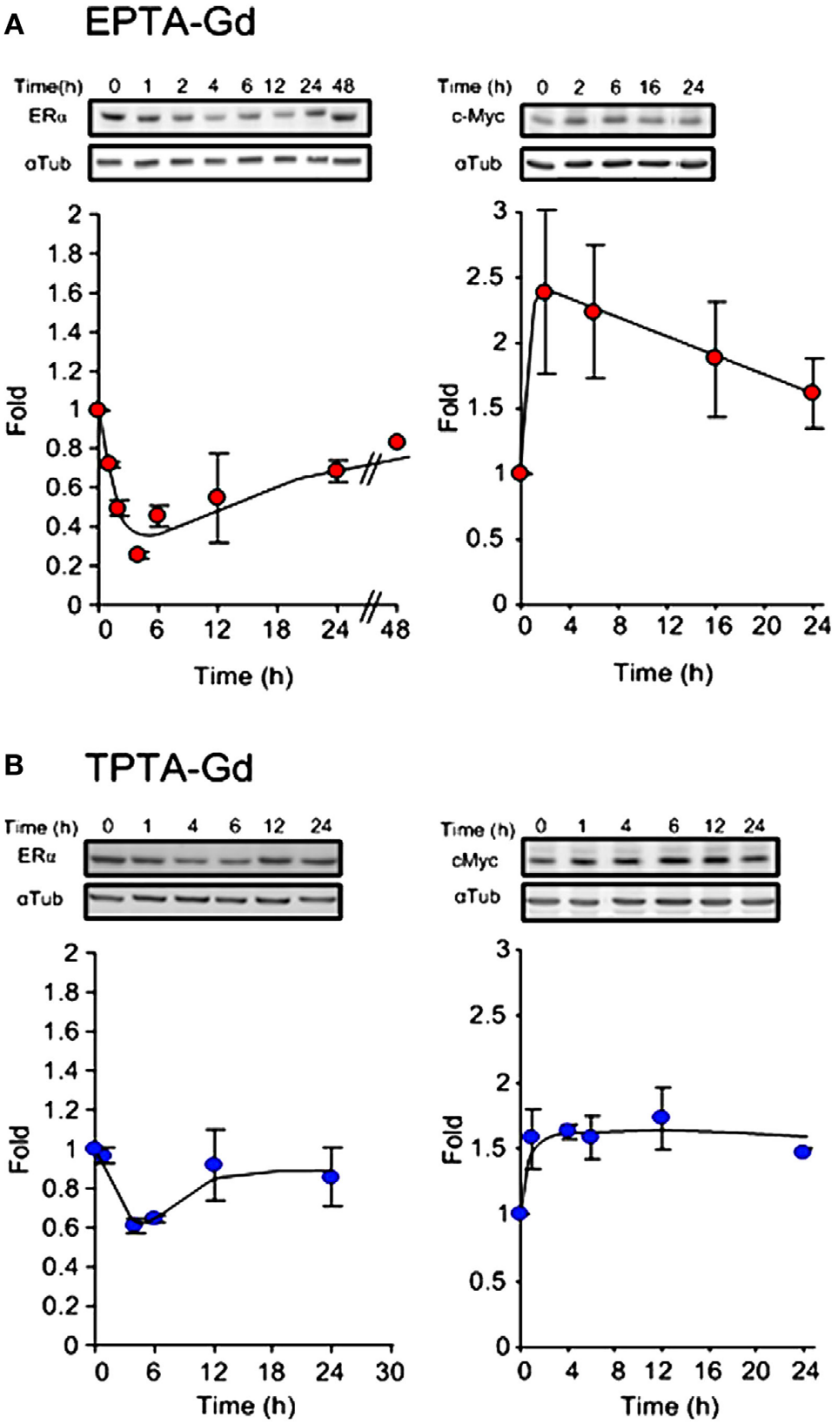
**Changes in the expression level of ERα (left) and cMyc (right) in MCF7 cells induced by EPTA-Gd (A) and TPTA-Gd (B) at a concentration of 5 μM**. In each panel, a representative blot is at the upper raw and the α-tubulin for normalization is at the bottom raw. The curve shows the fold change in expression levels, *n* = 2.

The ability of the two targeted probes to regulate specific estrogen-induced molecular changes was investigated in MCF7 cells. EPTA-Gd treatment induced a marked effect on cMyc and ERα expression level upregulating the level of cMyc by ~250% within 2 h and reducing ER level to 25% of its initial value at 4 h. Figure 6A shows the time course of the changes in cMyc and ERα expression induced by EPTA-Gd, obtained from two independent experiments. This time course is very similar to that reported for 17β-estradiol in the same type of cells (21). TPTA-Gd showed less pronounced effects than EPTA-Gd, yet cMyc expression increased to ~150% of its initial level and ER level decreased to 60% of its initial level (Figure 6B, two independent experiments). These specific induced activities demonstrated the agonistic activity of EPTA-Gd and TPTA-Gd in a mechanism similar to 17β-estradiol.

### *In Vivo* Interaction with ER

The *in vivo* interaction of EPTA-Gd and TPTA-Gd with ER was investigated in orthotopic breast cancer xenografts of ER-positive and ER-negative cells implanted in the same SCID mouse and were partly described in earlier publications (17, 18). Solid palpable tumors developed within a week after the implantation of cells into the mammary fat pad of the SCID mice. Both the ER-positive and ER-negative xenografts grew at a similar rate with no significant difference in their volume size during 22 days of growth (*n* = 10, paired *t*-test: *p* ≥ 0.9 in days 8, 11, 16, 18, and 22 after implantation of the cells), reaching at 22 days a mean size of 220 ± 65 mm^3^.

**Figure 7:**
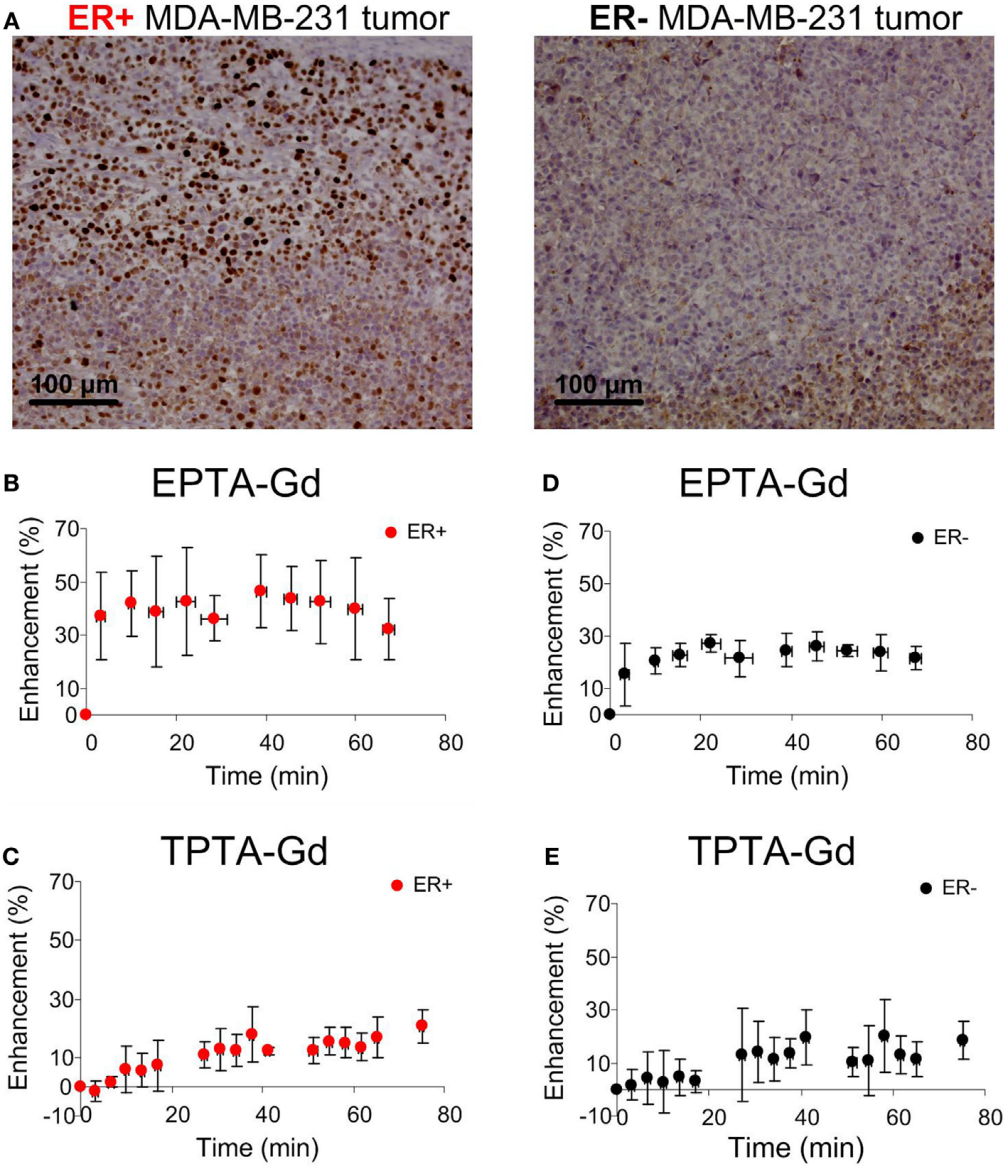
**Immunohistology staining of ERα (A) and MRI signal enhancement patterns induced by EPTA-Gd (B,D) and by TPTA-Gd (C,E) in ER-positive and ER-negative MDA-MB-231 human breast cancer xenografts**. The injected dose of EPTA-Gd and TPTA-Gd was 0.075 mmol/kg. The curves in **(B,D)** show mean ± SD of EPTA-Gd-induced enhancement (*n* = 5). The curves in **(C,E)** show mean ± SD of TPTA-Gd-induced enhancement (*n* = 4).

Immunostaining of the ER-positive xenografts showed high nuclear ER staining in viable regions, whereas in the ER-negative xenografts the staining was very low (Figure 7A). Quantitative analysis showed that the percentage of ER-stained cells in the ER-positive xenografts was more than 10-fold higher (71.0 ± 0.05%, *n* = 11) than in the ER-negative xenografts (2.12 ± 0.01%, *n* = 11). Similarly, the specific intensity index in the ER-positive xenografts was 10-fold higher (2.12 ± 0.01) than in the ER-negative xenografts (0.02 ± 0.08).

**Figure 8:**
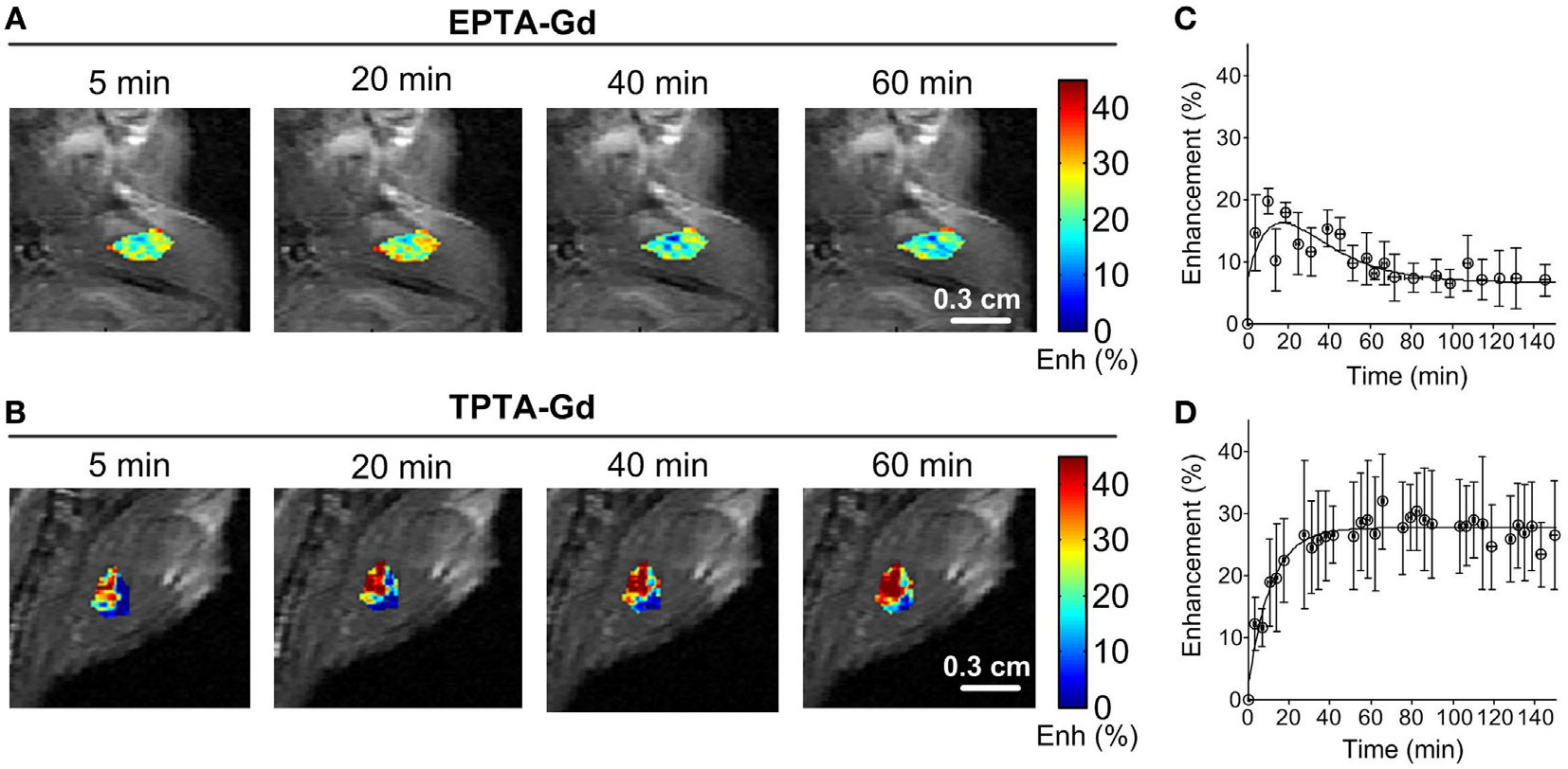
**Enhancement maps in muscle tissue 20 min after bolus injection of EPTA-Gd (A) and TPTA-Gd (B) into the tail vein of the mice and mean ± SD enhancement curves induced by EPTA-Gd [(C), *n* = 5] and TPTA-Gd [(D), *n* = 4]**. The injected dose of EPTA-Gd and TPTA-Gd was 0.075 mmol/kg. The enhancement maps are overlaid on the corresponding T1-weighted images.

Administration of EPTA-Gd and TPTA-Gd into the blood circulation caused enhancement of the water signal with different kinetic profiles for the ER-positive and ER-negative tumors, as shown in the time courses of the mean ± SD enhancement curves in Figures 7B–E. The EPTA-Gd enhancement profile in the tumors indicated fast wash-in followed by a plateau, with the enhancement in the ER-positive xenografts (reaching 43 ± 20% at 40 min postinjection) significantly higher than in the ER-negative xenografts (reaching 25 ± 5% at 40 min postinjection; *p* = 0.005, *n* = 9). In contrast, TPTA-Gd caused a low enhancement of 13 ± 2% at 40 min postinjection, which did not differ significantly from the enhancement in the ER-negative cells (*p* = 0.21, *n* = 4). As a reference to ER-negative tissue, we also monitored the enhancement induced by each agent in muscle tissues (Figure 8). The enhancement induced by EPTA-Gd in the muscle was low 15 ± 3% at 40 min postinjection (*n* = 9), with a wash-in followed by a marked washout phase (Figure 8C). In contrast, TPTA-Gd induced an appreciable enhancement in the muscle tissue (28 ± 5% at 40 min postinjection, *n* = 4), with no washout during the 140 min of the entire experiment (Figure 8D). These results indicated that the enhancement induced by EPTA-Gd in ER-positive tumors is significantly higher than in ER-negative tumors and in muscle tissue, however, TPTA-Gd exhibited similar and low enhancement in ER-positive and negative tumors and a specific interaction with muscle component(s). Since the PTA-part is identical in both EPTA-Gd and TPTA-Gd, the accumulation of TPTA-Gd in the muscle is most likely due to the interaction of a muscle component with the tamoxifen moiety.

### Competition Studies of EPTA-Gd with Tamoxifen

**Figure 9:**
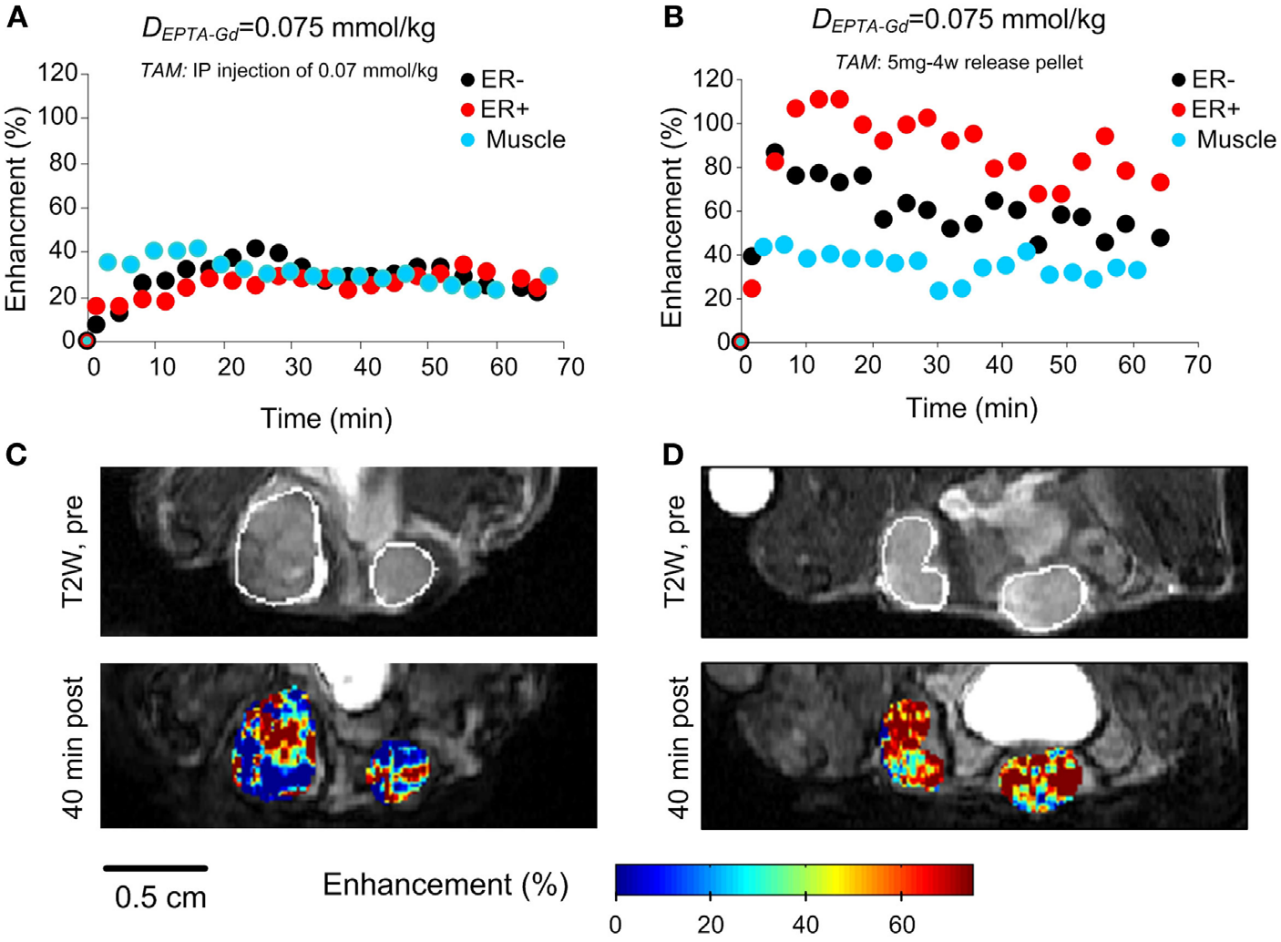
**EPTA-Gd induced enhancement patterns (A,B) and enhancement maps at 40 min (C,D) in ER-positive and ER-negative MDA-MB-231 xenografts treated with acute (1 h) and chronic (3 days) tamoxifen**. The anatomy of the xengorafts is demonstrated in the T2-weighted images **(C,D)**. The enhancement maps are overlaid on the corresponding T1-weighted images.

Investigation of the time course and extent of EPTA-Gd-induced enhancement showed that shortly after tamoxifen treatment (1 h), a similar enhancement of ~30% at 40 min postinjection was detected in ER-positive and ER-negative xenografts (*p* = 0.93, *n* = 4), as well as in muscle tissue (*p* = 0.43, *n* = 4) (Figures 9A,C). These results confirmed the binding of tamoxifen to ER and complete blocking of the interaction of EPTA-Gd to the ER.

Chronic treatment with tamoxifen for 3 days also caused the enhancement to be similar in ER-positive and ER-negative xenografts (*p* = 0.36, *n* = 4), confirming tamoxifen blockage of ER and inhibition of EPTA-Gd binding. However, the extent of maximum enhancement in the chronic treated xenografts was significantly higher (77 ± 16%) than that reached after acute, 1 h treatment with tamoxifen (28 ± 7%; *p* = 0.003, *n* = 4) (Figures 9B,D). This unexpected result indicated an effect of tamoxifen chronic treatment on tumors’ vascular function, increasing the microvascular transcapillary transfer rates in both ER-positive and ER-negative tumors by an hormonal independent mechanism.

## Discussion

The ER is a major prognostic biomarker in breast cancer and a valuable predictor of response to hormonal therapy. New molecular imaging techniques that will enable to detect ER presence and distribution *in vivo*, in both primary and metastatic cancer lesions, could improve accuracy and reproducibility of prognostic assessment and therapy management of breast cancer patients. In this study, we describe the interactions, biological activity, and imaging efficiency of two new paramagnetic ER-targeted CAs in human breast cancer cells and human breast cancer xenografts.

We first measured the ER binding of EPTA-Gd and TPTA-Gd in solution and then monitored the interaction of these probes with ER in human breast cancer cells. In solution, despite the bulky organometallic moiety at the 17α-position of 17β-estradiol or *trans* 4-position of tamoxifen (occupied by an hydroxyl group in 4-hydroxy tamoxifen), the binding affinity to the receptor was at the micromolar range, as was also shown previously for other organometallic-labeled estradiol derivatives (25–27). Although the affinity of both ER-targeted probes was two orders of magnitude lower than that of tamoxifen, it appeared to be sufficiently high for interacting and binding to ER in breast cancer cells and xenografts using micromolar concentrations. The MRI relaxation studies and fluorescence binding confirmed the binding inside the cells, most likely to nuclear ER. MRI T1-relaxation studies in ER-positive and negative cells of the same origin showed augmentation of the T1-relaxation rate and the T1 relaxivity in the ER-positive cells as compared to ER-negative cells, indicating entrance to the cells and nuclei and binding to the receptor. Measurements of T1 and T2 in breast cancer cells perfused with medium containing varying concentrations of the ER-targeted probes further confirmed interaction with ER. Generally, Gadolinium-based CAs will affect both T1- and T2-relaxation rates. However, in *in vivo* studies of DCE MRI, due to time limitations, gradient-echo relaxation weighted sequences are applied. The effect of the CA on the signal intensity in T1-weighted images is positive, namely, increase in signal, whereas in T2-weighted images it is negative, namely, signal reduction. An increase in the signal intensity can be detected more efficiently and accurately than the decrease in a T2-weighted image, particularly for a CA that is diffusing into the tissue and when the pre-contrast signal to noise ratio is low, as usually is the situation *in vivo*. Therefore, we predominantly concentrated on T1 measurements using a gradient-echo T1-weighted sequence in the *in vivo* studies.

Fluorescence-binding studies of the analogous paramagnetic agent EPTA-Eu provided a direct measure of the binding affinity in ER-positive cells and confirmed the capability of this agent to enter the cells and to bind to ER as in solution, with a micromolar-binding affinity. It should be noted that relaxation and fluorescence studies in ER-negative cells indicated the presence of non-specific interactions with cell components, but these interactions did not mask the binding and interaction in the ER-positive cells. The ER non-specific interactions of TPTA-Gd appeared to be higher than those of EPTA-Gd, making this agent less favorable as a targeted probe. It was not possible to predict in advance whether the binding of the new probes to ER in cells will modulate gene expression in a similar manner to that of 17β-estradiol or of tamoxifen. It was expected that the 17β-estradiol moiety of EPTA-Gd will induce agonistic activity upon binding to ER, whereas the tamoxifen moiety may exhibit antagonistic activity like tamoxifen. Testing the SERM activity of EPTA-Gd and TPTA-Gd was therefore performed in well-established ER responsive human breast cancer cell lines, MCF7 and T47D, in reference to the ER-negative MDA-MB-231 human breast cancer cells. We have investigated typical estrogen-modulated processes that are known to be inhibited by tamoxifen, such as cell proliferation, the expression level of cMyc, and the ER-expression level [Ref. (28–31) and references cited therein]. EPTA-Gd and TPTA-Gd exhibited estrogen-like agonistic effects, with EPTA-Gd mimicking closely estradiol activity and TPTA-Gd showing mild agonistic effects. Both new agents stimulated cell proliferation at a dose-dependent manner. This stimulation was specific to the presence of ER in the cells, as there was no effect on the growth of ER-negative MDA-MB-231 human breast cancer cells. They also induced the upregulation of cMyc and downregulation of ER, due to ER-enhanced degradation. The ability of these two new agents to induced typical estrogen-like activities clearly indicated their capability to enter the cells and nuclei and interact with the receptor to trigger specific estrogen-mediated responses. Furthermore, the E2-like activities demonstrated that the conjugated pyridine–Gd complex of EPTA-Gd did not alter the function of the 17β-estradiol moiety in inducing estrogenic agonistic-like activity on breast cancer cell, whereas the conjugated pyridine–Gd complex in TPTA-Gd modified the antagonistic function of the tamoxifen moiety yielding a mild agonistic activity.

The ability of EPTA-Gd to act agonistically was strongly supported by the X-ray crystalographic structure of the complex of EPTA-Eu with the ligand-binding domain of ER (19). Because of the chirality of C17 in the 17β-estradiol moiety and due to the rigid triple bond linking it with the PTA-Eu moiety, the orientation of the organometallic moiety is fixed within the ligand-binding cavity, being almost perpendicular to the flat face of 17β-estradiol and pointing toward helix 7 in an opposite direction to helix 12. Consequently, the conformation of the 17β-estradiol moiety of EPTA-Gd in the ER-binding cavity is almost equivalent to that of free 17β-estradiol and the paramagnetic PTA-Gd moiety causes uncoiling of helices 7 and 8, but does not perturb the agonistic activity determined by helix 12 conformation.

As TPTA-Gd is based on a tamoxifen moiety, it was not clear which conformation it will adopt in the ER-binding cavity and how it will modulate the activity. If the 4-[2 -(dimethyl amino) ethoxy] group of the tamoxifen moiety would have been aligned in the ligand-binding cavity as tamoxifen, it could change helix 12 conformation in a similar manner to the change induced by 4-hydroxy tamoxifen (32), leading to antiestrogen activity. However, the rigid triple bond in the 4-position and the flexibility of tamoxifen structure could lead to an enlargement and distortion of the binding cavity in a similar way to that obtained with EPTA-Gd directing the PTA-Gd moiety toward helices 7 and 8 and accommodating the dimethylamino ethoxy group in a conformation, which does not perturb significantly helix 12 conformation. This hypothesis is in accord with recent X-ray studies suggesting that the flexibility and plasticity of the entire ligand-binding cavity of ER allows expanding of the cavity space in different directions, depending on the chemical nature of the bound ligand (33–35). The well-defined structure by X-ray crystallography provides direct evidence on the ability of a ligand to interact with ER in the solid state and induce agonistic or antagonistic activity. However, it should be noted that conformational changes of ER in the tissue environment, specifically in the nucleus, may alter the interaction between the ligand and the receptor and modify the induced activity.

The main purpose of the *in vivo* studies was to evaluate the ability of the two ER-targeted CAs to demonstrate augmented enhancement upon binding to ER, thereby detecting ER-positive tumors. Indeed, EPTA-Gd interacted with ER as expected from the cell culture studies and specifically augmented significantly the MRI signal in the ER-positive xenografts as compared to the ER-negative xenografts and to muscle tissue. The muscle enhancement was low and also decayed after ~20 min as expected for molecules that do not show specific interaction with extracellular or intracellular components and are cleared out through the kidneys (17). Furthermore, the inability of EPTA-Gd to induce enhancement when ER was blocked by tamoxifen served to prove its selective binding to free ER. In contrast, the interaction and enhancement patterns induced by TPTA-Gd could not be predicted from the breast cancer cell culture studies, as this probe strongly enhanced *in vivo* muscle tissues, demonstrating a dominant non-ER-specific binding to muscle components. This non-specific interaction is most likely determined by the tamoxifen moiety as the paramagnetic moiety is the same in EPTA-Gd and TPTA-Gd. It was previously shown that in addition to its high-affinity binding to the ER, tamoxifen binds with high affinity to microsomal antiestrogen-binding site (AEBS) and inhibits with a micromolar efficiency, protein kinase C (PKC), calmodulin (CaM)-dependent enzymes, and Acyl coenzyme A: cholesterol acyltransferase (ACAT) (36–38).

An additional ER-independent effect of tamoxifen was revealed in the competition studies of EPTA-Gd and tamoxifen. Acute tamoxifen treatment for 1 h did not affect the vascular function of the ER-positive and negative xenografts. However, chronic treatment for 3 days caused in both types of xenografts a higher transcapillary transfer and augmented enhancement suggesting specific tamoxifen-induced interaction with tumor endothelial cells that change the vascular permeability. It was previously demonstrated that chronic tamoxifen treatment for several days increased the transcapillary transfer of MCF7 xenografts in nude mice (39). It was also shown by Blackwell et al. (40) that tamoxifen exerts significant inhibition of angiogenesis in an ER-independent mechanism. The effects on the vascular function during chronic treatment with tamoxifen could be indirect, such as increased hypoxia and consequently upregulation of VEGF expression level, but we cannot exclude direct interaction with endothelial cells.

Of specific interest was the effect of EPTA-Gd on ER level. The amount of ER in cells is controlled by a balance between its synthesis and degradation and is influenced by the nature of the bound ligand. Like estrogen treatment (21), EPTA-Gd induced a marked decrease in ER within few hours, which slowly returned to steady state level at 48 h posttreatment. This temporal change in ER level are directly associated with the functional response of ER and reflects estrogen responsiveness in general. Further studies are required for extending the use of EPTA-Gd to monitor temporal changes in ER level and characterize ER functional activity. Moreover, it was recently indicated that the shape of an estrogenic ligand programs the conformation of the ER complex, which, in turn, can modulate the activity of estrogen-induced apoptosis (41). The mechanism of estrogen-induced apoptosis has been associated with the positive response to treatment with estradiol, or with partial ER agonists of ER-positive breast cancers resistant to long-term estrogen deprivation (for example, by tamoxifen) (42, 43).

The ability of partial ER agonists to induce apoptosis in ER-positive cells suggests that the clinical application of EPTA-Gd may not cause any harm to breast cancer patients with ER-positive tumors and could even be effective by inducing apoptosis. Further studies investigating whether EPTA-Gd can induce apoptosis may lead to its development as a dual-targeted ER probe for imaging and treatment, particularly of tamoxifen-resistant ER-positive cancers. Clearly, although EPTA-Gd was found to be non-toxic to mice, at the doses administered in this study, and to be cleared out into the urine, any future clinical utilization will require controlled toxicity studies in humans. Thus far, new MRI probes targeted to the progesterone receptor were also synthesized, demonstrating specific enhancement in progesterone – positive cancer cells and animal model tumors (44–47). Currently, however, the only imaging approach for visual assessment and quantitative measurement of steroid hormone receptors in humans have focused predominately on ^18^F-based radiopharmaceuticals and PET, in combination with computed tomography (48).

In summary, EPTA-Gd and TPTA-Gd were found to act as SERMs with partial agonistic activities, inducing proliferation of estrogen responsive cells and modulating gene expression levels of ER and cMyc. Both agents entered the cells and augmenting the T1-relaxation rate of the water in their surrounding through binding to ER. However, only EPTA-Gd was found to be an adequate ER-targeted probe *in vivo* as TPTA-Gd exhibited non-specific interaction with cell components other than ER, particularly in muscle tissue, that dominated its ability to induce MRI contrast in ER-positive tumors.

## Author Contributions

AP performed the entire acquisition, analysis, and interpretation of the data in this work, worked on drafting this manuscript and preparing the illustrations, including approval of the final version, and accounting for all aspects of the work in ensuring that questions related to the accuracy or integrity of any part of the work are appropriately investigated and resolved. HD is responsible for the conception of the work and for the design of the experiments and the interpretation of the data, revising the manuscript and adding content of intellectual importance including approving the final version, and accounting for all aspects of the work in ensuring that questions related to the accuracy or integrity of any part of the work are appropriately investigated and resolved.

## Conflict of Interest Statement

The research was conducted in the absence of any commercial or financial relationships that could be construed as a potential conflict of interest.
